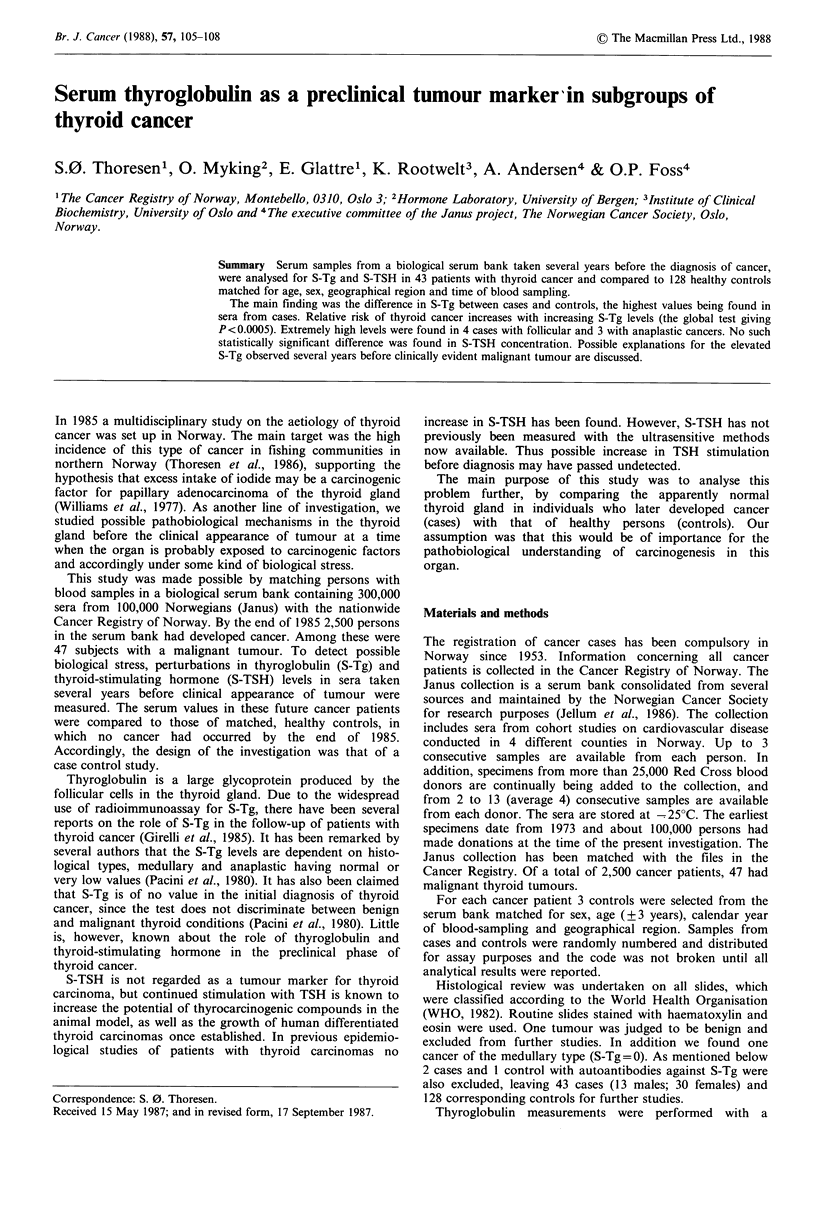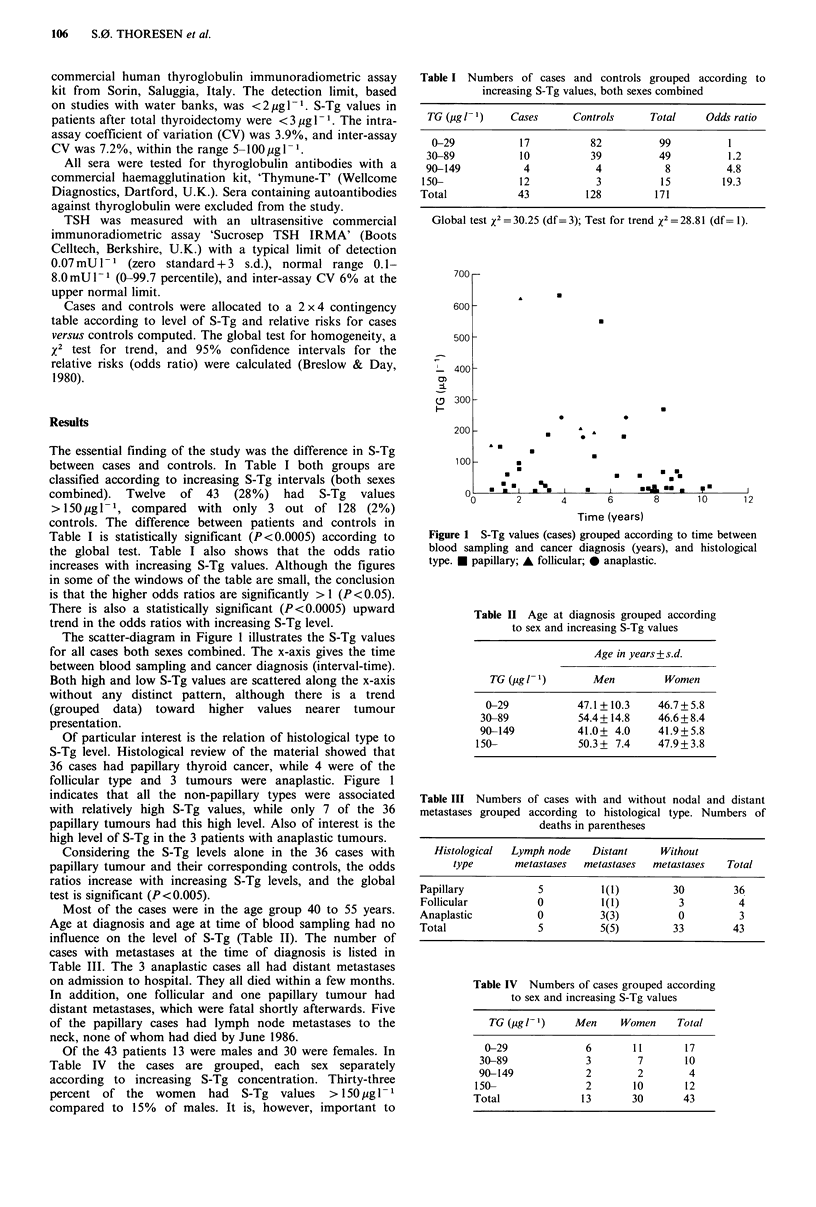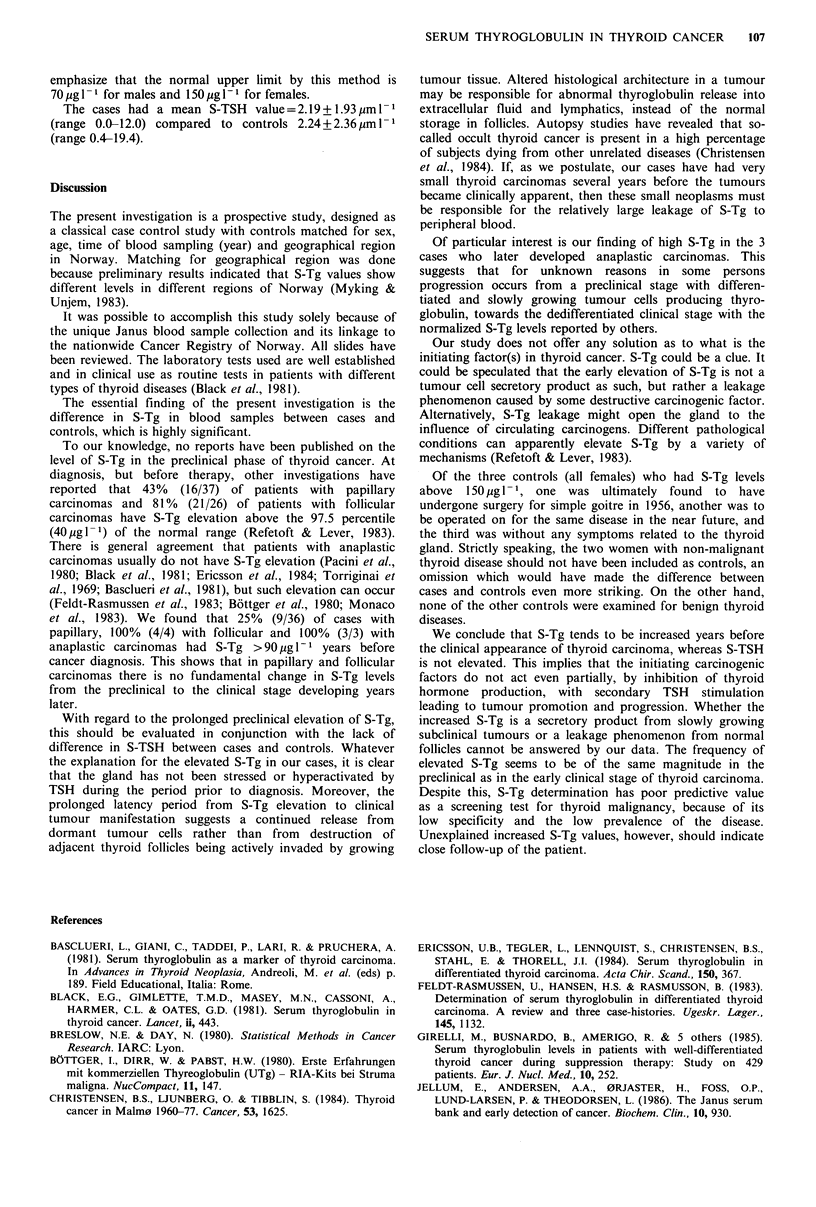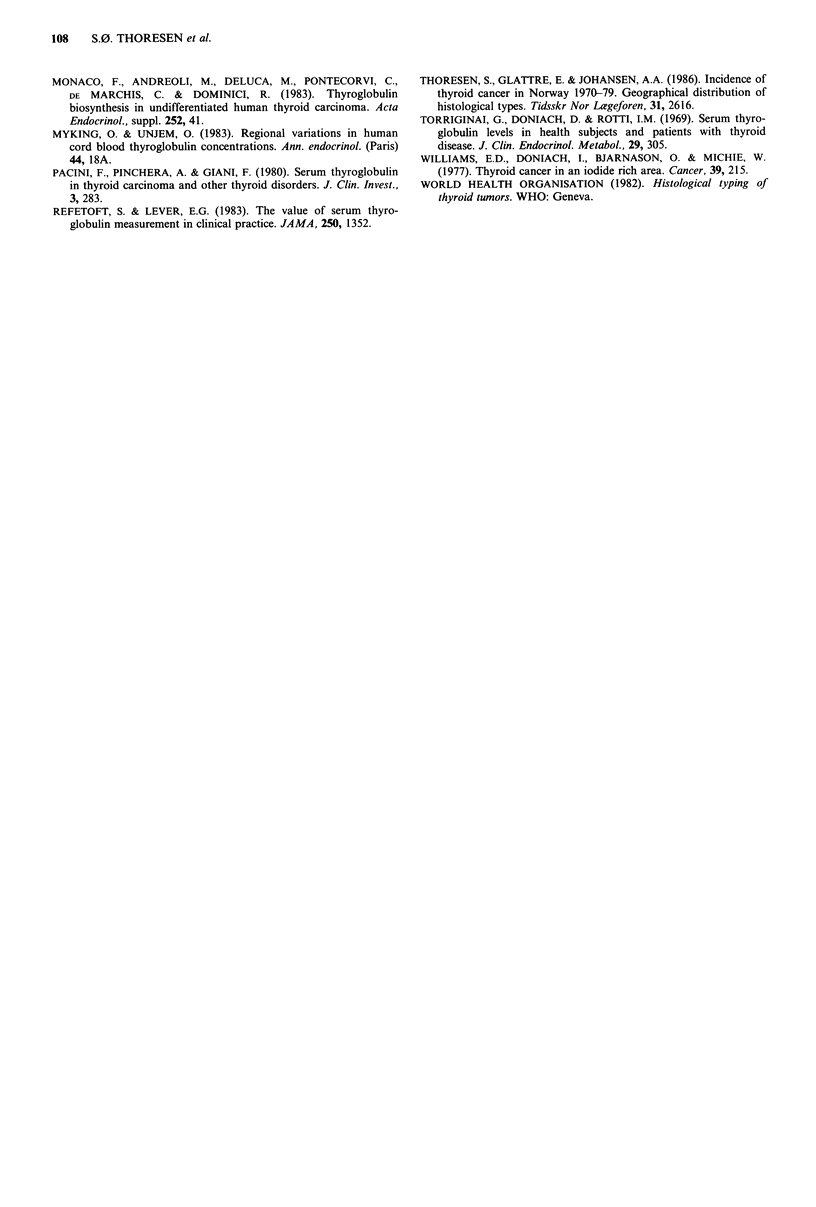# Serum thyroglobulin as a preclinical tumour marker in subgroups of thyroid cancer.

**DOI:** 10.1038/bjc.1988.19

**Published:** 1988-01

**Authors:** S. O. Thoresen, O. Myking, E. Glattre, K. Rootwelt, A. Andersen, O. P. Foss

**Affiliations:** Cancer Registry of Norway, Montebello.

## Abstract

Serum samples from a biological serum bank taken several years before the diagnosis of cancer, were analysed for S-Tg and S-TSH in 43 patients with thyroid cancer and compared to 128 healthy controls matched for age, sex, geographical region and time of blood sampling. The main finding was the difference in S-Tg between cases and controls, the highest values being found in sera from cases. Relative risk of thyroid cancer increases with increasing S-Tg levels (the global test giving P less than 0.0005). Extremely high levels were found in 4 cases with follicular and 3 with anaplastic cancers. No such statistically significant difference was found in S-TSH concentration. Possible explanations for the elevated S-Tg observed several years before clinically evident malignant tumour are discussed.


Br. J. Cancer (1988), 57, 105–108 © The Macmillan Press Ltd., 1988

### Serum thyroglobulin as a preclinical tumour marker in subgroups of thyroid cancer

S.Ø. Thoresen[1], O. Myking[2], E. Glattre[1], K. Rootwelt[3], A. Andersen[4] & O.P. Foss[4]

[1]The Cancer Registry of Norway, Montebello, 0310, Oslo 3; [2]Hormone Laboratory, University of Bergen; [3]Institute of Clinical Biochemistry, University of Oslo and [4]The executive committee of the Janus project, The Norwegian Cancer Society, Oslo, Norway.

**Summary** Serum samples from a biological serum bank taken several years before the diagnosis of cancer, were analysed for S-Tg and S-TSH in 43 patients with thyroid cancer and compared to 128 healthy controls matched for age, sex, geographical region and time of blood sampling.

The main finding was the difference in S-Tg between cases and controls, the highest values being found in sera from cases. Relative risk of thyroid cancer increases with increasing S-Tg levels (the global test giving $P<0.0005$). Extremely high levels were found in 4 cases with follicular and 3 with anaplastic cancers. No such statistically significant difference was found in S-TSH concentration. Possible explanations for the elevated S-Tg observed several years before clinically evident malignant tumour are discussed.

In 1985 a multidisciplinary study on the aetiology of thyroid cancer was set up in Norway. The main target was the high incidence of this type of cancer in fishing communities in northern Norway (Thoresen *et al.*, 1986), supporting the hypothesis that excess intake of iodide may be a carcinogenic factor for papillary adenocarcinoma of the thyroid gland (Williams *et al.*, 1977). As another line of investigation, we studied possible pathobiological mechanisms in the thyroid gland before the clinical appearance of tumour at a time when the organ is probably exposed to carcinogenic factors and accordingly under some kind of biological stress.

This study was made possible by matching persons with blood samples in a biological serum bank containing 300,000 sera from 100,000 Norwegians (Janus) with the nationwide Cancer Registry of Norway. By the end of 1985 2,500 persons in the serum bank had developed cancer. Among these were 47 subjects with a malignant tumour. To detect possible biological stress, perturbations in thyroglobulin (S-Tg) and thyroid-stimulating hormone (S-TSH) levels in sera taken several years before clinical appearance of tumour were measured. The serum values in these future cancer patients were compared to those of matched, healthy controls, in which no cancer had occurred by the end of 1985. Accordingly, the design of the investigation was that of a case control study.

Thyroglobulin is a large glycoprotein produced by the follicular cells in the thyroid gland. Due to the widespread use of radioimmunoassay for S-Tg, there have been several reports on the role of S-Tg in the follow-up of patients with thyroid cancer (Girelli *et al.*, 1985). It has been remarked by several authors that the S-Tg levels are dependent on histological types, medullary and anaplastic having normal or very low values (Pacini *et al.*, 1980). It has also been claimed that S-Tg is of no value in the initial diagnosis of thyroid cancer, since the test does not discriminate between benign and malignant thyroid conditions (Pacini *et al.*, 1980). Little is, however, known about the role of thyroglobulin and thyroid-stimulating hormone in the preclinical phase of thyroid cancer.

S-TSH is not regarded as a tumour marker for thyroid carcinoma, but continued stimulation with TSH is known to increase the potential of thyrocarcinogenic compounds in the animal model, as well as the growth of human differentiated thyroid carcinomas once established. In previous epidemiological studies of patients with thyroid carcinomas no increase in S-TSH has been found. However, S-TSH has not previously been measured with the ultrasensitive methods now available. Thus possible increase in TSH stimulation before diagnosis may have passed undetected.

The main purpose of this study was to analyse this problem further, by comparing the apparently normal thyroid gland in individuals who later developed cancer (cases) with that of healthy persons (controls). Our assumption was that this would be of importance for the pathobiological understanding of carcinogenesis in this organ.

### Materials and methods

The registration of cancer cases has been compulsory in Norway since 1953. Information concerning all cancer patients is collected in the Cancer Registry of Norway. The Janus collection is a serum bank consolidated from several sources and maintained by the Norwegian Cancer Society for research purposes (Jellum *et al.*, 1986). The collection includes sera from cohort studies on cardiovascular disease conducted in 4 different counties in Norway. Up to 3 consecutive samples are available from each person. In addition, specimens from more than 25,000 Red Cross blood donors are continually being added to the collection, and from 2 to 13 (average 4) consecutive samples are available from each donor. The sera are stored at $-25°C$. The earliest specimens date from 1973 and about 100,000 persons had made donations at the time of the present investigation. The Janus collection has been matched with the files in the Cancer Registry. Of a total of 2,500 cancer patients, 47 had malignant thyroid tumours.

For each cancer patient 3 controls were selected from the serum bank matched for sex, age ($\pm 3$ years), calendar year of blood-sampling and geographical region. Samples from cases and controls were randomly numbered and distributed for assay purposes and the code was not broken until all analytical results were reported.

Histological review was undertaken on all slides, which were classified according to the World Health Organisation (WHO, 1982). Routine slides stained with haematoxylin and eosin were used. One tumour was judged to be benign and excluded from further studies. In addition we found one cancer of the medullary type (S-Tg=0). As mentioned below 2 cases and 1 control with autoantibodies against S-Tg were also excluded, leaving 43 cases (13 males; 30 females) and 128 corresponding controls for further studies.

Thyroglobulin measurements were performed with a

Correspondence: S. Ø. Thoresen.
Received 15 May 1987; and in revised form, 17 September 1987.

commercial human thyroglobulin immunoradiometric assay kit from Sorin, Saluggia, Italy. The detection limit, based on studies with water banks, was $<2\,\mu g\,l^{-1}$. S-Tg values in patients after total thyroidectomy were $<3\,\mu g\,l^{-1}$. The intra-assay coefficient of variation (CV) was 3.9%, and inter-assay CV was 7.2%, within the range $5$–$100\,\mu g\,l^{-1}$.

All sera were tested for thyroglobulin antibodies with a commercial haemagglutination kit, 'Thymune-T' (Wellcome Diagnostics, Dartford, U.K.). Sera containing autoantibodies against thyroglobulin were excluded from the study.

TSH was measured with an ultrasensitive commercial immunoradiometric assay 'Sucrosep TSH IRMA' (Boots Celltech, Berkshire, U.K.) with a typical limit of detection $0.07\,mU\,l^{-1}$ (zero standard + 3 s.d.), normal range 0.1–$8.0\,mU\,l^{-1}$ (0–99.7 percentile), and inter-assay CV 6% at the upper normal limit.

Cases and controls were allocated to a $2\times4$ contingency table according to level of S-Tg and relative risks for cases *versus* controls computed. The global test for homogeneity, a $\chi^2$ test for trend, and 95% confidence intervals for the relative risks (odds ratio) were calculated (Breslow & Day, 1980).

### Results

The essential finding of the study was the difference in S-Tg between cases and controls. In Table I both groups are classified according to increasing S-Tg intervals (both sexes combined). Twelve of 43 (28%) had S-Tg values $>150\,\mu g\,l^{-1}$, compared with only 3 out of 128 (2%) controls. The difference between patients and controls in Table I is statistically significant ($P<0.0005$) according to the global test. Table I also shows that the odds ratio increases with increasing S-Tg values. Although the figures in some of the windows of the table are small, the conclusion is that the higher odds ratios are significantly $>1$ ($P<0.05$). There is also a statistically significant ($P<0.0005$) upward trend in the odds ratios with increasing S-Tg level.

The scatter-diagram in Figure 1 illustrates the S-Tg values for all cases both sexes combined. The x-axis gives the time between blood sampling and cancer diagnosis (interval-time). Both high and low S-Tg values are scattered along the x-axis without any distinct pattern, although there is a trend (grouped data) toward higher values nearer tumour presentation.

Of particular interest is the relation of histological type to S-Tg level. Histological review of the material showed that 36 cases had papillary thyroid cancer, while 4 were of the follicular type and 3 tumours were anaplastic. Figure 1 indicates that all the non-papillary types were associated with relatively high S-Tg values, while only 7 of the 36 papillary tumours had this high level. Also of interest is the high level of S-Tg in the 3 patients with anaplastic tumours.

Considering the S-Tg levels alone in the 36 cases with papillary tumour and their corresponding controls, the odds ratios increase with increasing S-Tg levels, and the global test is significant ($P<0.005$).

Most of the cases were in the age group 40 to 55 years. Age at diagnosis and age at time of blood sampling had no influence on the level of S-Tg (Table II). The number of cases with metastases at the time of diagnosis is listed in Table III. The 3 anaplastic cases all had distant metastases on admission to hospital. They all died within a few months. In addition, one follicular and one papillary tumour had distant metastases, which were fatal shortly afterwards. Five of the papillary cases had lymph node metastases to the neck, none of whom had died by June 1986.

Of the 43 patients 13 were males and 30 were females. In Table IV the cases are grouped, each sex separately according to increasing S-Tg concentration. Thirty-three percent of the women had S-Tg values $>150\,\mu g\,l^{-1}$ compared to 15% of males. It is, however, important to

**Table I** Numbers of cases and controls grouped according to increasing S-Tg values, both sexes combined

| *TG* ($\mu g\,l^{-1}$) | *Cases* | *Controls* | *Total* | *Odds ratio* |
|---|---|---|---|---|
| 0–29 | 17 | 82 | 99 | 1 |
| 30–89 | 10 | 39 | 49 | 1.2 |
| 90–149 | 4 | 4 | 8 | 4.8 |
| 150– | 12 | 3 | 15 | 19.3 |
| Total | 43 | 128 | 171 | |

Global test $\chi^2=30.25$ (df = 3); Test for trend $\chi^2=28.81$ (df = 1).

**Figure 1** S-Tg values (cases) grouped according to time between blood sampling and cancer diagnosis (years), and histological type. ■ papillary; ▲ follicular; ● anaplastic.

**Table II** Age at diagnosis grouped according to sex and increasing S-Tg values

| | *Age in years* ± *s.d.* | |
|---|---|---|
| *TG* ($\mu g\,l^{-1}$) | *Men* | *Women* |
| 0–29 | 47.1 ± 10.3 | 46.7 ± 5.8 |
| 30–89 | 54.4 ± 14.8 | 46.6 ± 8.4 |
| 90–149 | 41.0 ± 4.0 | 41.9 ± 5.8 |
| 150– | 50.3 ± 7.4 | 47.9 ± 3.8 |

**Table III** Numbers of cases with and without nodal and distant metastases grouped according to histological type. Numbers of deaths in parentheses

| *Histological type* | *Lymph node metastases* | *Distant metastases* | *Without metastases* | *Total* |
|---|---|---|---|---|
| Papillary | 5 | 1(1) | 30 | 36 |
| Follicular | 0 | 1(1) | 3 | 4 |
| Anaplastic | 0 | 3(3) | 0 | 3 |
| Total | 5 | 5(5) | 33 | 43 |

**Table IV** Numbers of cases grouped according to sex and increasing S-Tg values

| *TG* ($\mu g\,l^{-1}$) | *Men* | *Women* | *Total* |
|---|---|---|---|
| 0–29 | 6 | 11 | 17 |
| 30–89 | 3 | 7 | 10 |
| 90–149 | 2 | 2 | 4 |
| 150– | 2 | 10 | 12 |
| Total | 13 | 30 | 43 |

emphasize that the normal upper limit by this method is $70 \mu g l^{-1}$ for males and $150 \mu g l^{-1}$ for females.

The cases had a mean S-TSH value $= 2.19 \pm 1.93 \mu m l^{-1}$ (range 0.0–12.0) compared to controls $2.24 \pm 2.36 \mu m l^{-1}$ (range 0.4–19.4).

### Discussion

The present investigation is a prospective study, designed as a classical case control study with controls matched for sex, age, time of blood sampling (year) and geographical region in Norway. Matching for geographical region was done because preliminary results indicated that S-Tg values show different levels in different regions of Norway (Myking & Unjem, 1983).

It was possible to accomplish this study solely because of the unique Janus blood sample collection and its linkage to the nationwide Cancer Registry of Norway. All slides have been reviewed. The laboratory tests used are well established and in clinical use as routine tests in patients with different types of thyroid diseases (Black *et al.*, 1981).

The essential finding of the present investigation is the difference in S-Tg in blood samples between cases and controls, which is highly significant.

To our knowledge, no reports have been published on the level of S-Tg in the preclinical phase of thyroid cancer. At diagnosis, but before therapy, other investigations have reported that 43% (16/37) of patients with papillary carcinomas and 81% (21/26) of patients with follicular carcinomas have S-Tg elevation above the 97.5 percentile ($40 \mu g l^{-1}$) of the normal range (Refetoft & Lever, 1983). There is general agreement that patients with anaplastic carcinomas usually do not have S-Tg elevation (Pacini *et al.*, 1980; Black *et al.*, 1981; Ericsson *et al.*, 1984; Torriginai *et al.*, 1969; Bascliueri *et al.*, 1981), but such elevation can occur (Feldt-Rasmussen *et al.*, 1983; Böttger *et al.*, 1980; Monaco *et al.*, 1983). We found that 25% (9/36) of cases with papillary, 100% (4/4) with follicular and 100% (3/3) with anaplastic carcinomas had S-Tg $> 90 \mu g l^{-1}$ years before cancer diagnosis. This shows that in papillary and follicular carcinomas there is no fundamental change in S-Tg levels from the preclinical to the clinical stage developing years later.

With regard to the prolonged preclinical elevation of S-Tg, this should be evaluated in conjunction with the lack of difference in S-TSH between cases and controls. Whatever the explanation for the elevated S-Tg in our cases, it is clear that the gland has not been stressed or hyperactivated by TSH during the period prior to diagnosis. Moreover, the prolonged latency period from S-Tg elevation to clinical tumour manifestation suggests a continued release from dormant tumour cells rather than from destruction of adjacent thyroid follicles being actively invaded by growing tumour tissue. Altered histological architecture in a tumour may be responsible for abnormal thyroglobulin release into extracellular fluid and lymphatics, instead of the normal storage in follicles. Autopsy studies have revealed that so-called occult thyroid cancer is present in a high percentage of subjects dying from other unrelated diseases (Christensen *et al.*, 1984). If, as we postulate, our cases have had very small thyroid carcinomas several years before the tumours became clinically apparent, then these small neoplasms must be responsible for the relatively large leakage of S-Tg to peripheral blood.

Of particular interest is our finding of high S-Tg in the 3 cases who later developed anaplastic carcinomas. This suggests that for unknown reasons in some persons progression occurs from a preclinical stage with differentiated and slowly growing tumour cells producing thyroglobulin, towards the dedifferentiated clinical stage with the normalized S-Tg levels reported by others.

Our study does not offer any solution as to what is the initiating factor(s) in thyroid cancer. S-Tg could be a clue. It could be speculated that the early elevation of S-Tg is not a tumour cell secretory product as such, but rather a leakage phenomenon caused by some destructive carcinogenic factor. Alternatively, S-Tg leakage might open the gland to the influence of circulating carcinogens. Different pathological conditions can apparently elevate S-Tg by a variety of mechanisms (Refetoft & Lever, 1983).

Of the three controls (all females) who had S-Tg levels above $150 \mu g l^{-1}$, one was ultimately found to have undergone surgery for simple goitre in 1956, another was to be operated on for the same disease in the near future, and the third was without any symptoms related to the thyroid gland. Strictly speaking, the two women with non-malignant thyroid disease should not have been included as controls, an omission which would have made the difference between cases and controls even more striking. On the other hand, none of the other controls were examined for benign thyroid diseases.

We conclude that S-Tg tends to be increased years before the clinical appearance of thyroid carcinoma, whereas S-TSH is not elevated. This implies that the initiating carcinogenic factors do not act even partially, by inhibition of thyroid hormone production, with secondary TSH stimulation leading to tumour promotion and progression. Whether the increased S-Tg is a secretory product from slowly growing subclinical tumours or a leakage phenomenon from normal follicles cannot be answered by our data. The frequency of elevated S-Tg seems to be of the same magnitude in the preclinical as in the early clinical stage of thyroid carcinoma. Despite this, S-Tg determination has poor predictive value as a screening test for thyroid malignancy, because of its low specificity and the low prevalence of the disease. Unexplained increased S-Tg values, however, should indicate close follow-up of the patient.

### References

BASCLUERI, L., GIANI, C., TADDEI, P., LARI, R. & PRUCHERA, A. (1981). Serum thyroglobulin as a marker of thyroid carcinoma. In *Advances in Thyroid Neoplasia*, Andreoli, M. *et al.* (eds) p. 189. Field Educational, Italia: Rome.

BLACK, E.G., GIMLETTE, T.M.D., MASEY, M.N., CASSONI, A., HARMER, C.L. & OATES, G.D. (1981). Serum thyroglobulin in thyroid cancer. *Lancet*, **ii**, 443.

BRESLOW, N.E. & DAY, N. (1980). *Statistical Methods in Cancer Research*. IARC: Lyon.

BÖTTGER, I., DIRR, W. & PABST, H.W. (1980). Erste Erfahrungen mit kommerziellen Thyreoglobulin (UTg) – RIA-Kits bei Struma maligna. *NucCompact*, **11**, 147.

CHRISTENSEN, B.S., LJUNBERG, O. & TIBBLIN, S. (1984). Thyroid cancer in Malmø 1960–77. *Cancer*, **53**, 1625.

ERICSSON, U.B., TEGLER, L., LENNQUIST, S., CHRISTENSEN, B.S., STAHL, E. & THORELL, J.I. (1984). Serum thyroglobulin in differentiated thyroid carcinoma. *Acta Chir. Scand.*, **150**, 367.

FELDT-RASMUSSEN, U., HANSEN, H.S. & RASMUSSON, B. (1983). Determination of serum thyroglobulin in differentiated thyroid carcinoma. A review and three case-histories. *Ugeskr. Læger.*, **145**, 1132.

GIRELLI, M., BUSNARDO, B., AMERIGO, R. & 5 others (1985). Serum thyroglobulin levels in patients with well-differentiated thyroid cancer during suppression therapy: Study on 429 patients. *Eur. J. Nucl. Med.*, **10**, 252.

JELLUM, E., ANDERSEN, A.A., ØRJASTER, H., FOSS, O.P., LUND-LARSEN, P. & THEODORSEN, L. (1986). The Janus serum bank and early detection of cancer. *Biochem. Clin.*, **10**, 930.

MONACO, F., ANDREOLI, M., DELUCA, M., PONTECORVI, C., DE MARCHIS, C. & DOMINICI, R. (1983). Thyroglobulin biosynthesis in undifferentiated human thyroid carcinoma. *Acta Endocrinol.*, suppl. **252**, 41.

MYKING, O. & UNJEM, O. (1983). Regional variations in human cord blood thyroglobulin concentrations. *Ann. endocrinol.* (Paris) **44**, 18A.

PACINI, F., PINCHERA, A. & GIANI, F. (1980). Serum thyroglobulin in thyroid carcinoma and other thyroid disorders. *J. Clin. Invest.*, **3**, 283.

REFETOFT, S. & LEVER, E.G. (1983). The value of serum thyroglobulin measurement in clinical practice. *JAMA*, **250**, 1352.

THORESEN, S., GLATTRE, E. & JOHANSEN, A.A. (1986). Incidence of thyroid cancer in Norway 1970–79. Geographical distribution of histological types. *Tidsskr Nor Lægeforen*, **31**, 2616.

TORRIGINAI, G., DONIACH, D. & ROTTI, I.M. (1969). Serum thyroglobulin levels in health subjects and patients with thyroid disease. *J. Clin. Endocrinol. Metabol.*, **29**, 305.

WILLIAMS, E.D., DONIACH, I., BJARNASON, O. & MICHIE, W. (1977). Thyroid cancer in an iodide rich area. *Cancer*, **39**, 215.

WORLD HEALTH ORGANISATION (1982). *Histological typing of thyroid tumors*. WHO: Geneva.